# A National Snapshot of Social Determinants of Health Documentation in Emergency Departments

**DOI:** 10.5811/westjem.58149

**Published:** 2023-07-12

**Authors:** Caitlin R. Ryus, Alexander T. Janke, Rachel L. Granovsky, Michael A. Granovsky

**Affiliations:** * Yale School of Medicine, Department of Emergency Medicine, New Haven, Connecticut; † University of California San Francisco, Institute for Health and Aging, San Francisco, California; ‡ LogixHealth, Bedford, Massachusetts; § VA HSR&D Center for the Study of Healthcare Innovation, Implementation, & Policy/Institute for Healthcare Policy and Innovation, University of Michigan, Ann Arbor, Michigan

## Abstract

**Introduction:**

Documentation and measurement of social determinants of health (SDoH) are critical to clinical care and to healthcare delivery system reforms targeting health equity. The SDoH are codified in the *International Classification of Disease 10^th^ Rev* (ICD-10) Z codes. However, Z codes are listed in only 1–2% of inpatient charts. Little is known about the frequency of Z code utilization specifically among emergency department (ED) patient populations nationally.

**Methods:**

This was a repeated cross-sectional analysis of ED visit data in the United States from the Nationwide Emergency Department Sample from 2016–2019. We characterized the use of Z codes and described associations between Z code use and patient- and hospital-level factors including the following: age; gender; race; insurance status; ED disposition; ED size; hospital urban-rural status; ownership; and clinical conditions. We calculated unadjusted odds ratios for likelihood of Z code reporting for each ED visit.

**Results:**

Of approximately 140 million ED visits per year, 0.65% had an associated Z code in 2016, rising to 1.17% by 2019. Visits were more likely to have an associated Z code for adults age <65, male, Black, Medicaid or self-pay patients, and patients admitted to the hospital. Larger EDs, those in metropolitan areas, academic centers, and government-run hospitals were more likely to report Z codes. The most commonly associated clinical conditions were as follows: schizophrenia spectrum and other psychotic disorders; depressive disorder; and alcohol-related disorders.

**Conclusion:**

There is a paucity of Z code documentation in the health records of ED patients, although use is uptrending. Further research is warranted to better understand the drivers of clinicians’ use of Z codes and to improve on their utility.

## INTRODUCTION

Documentation and measurement of social determinants of health (SDoH) are critical to high-quality clinical care, population health research, and to healthcare delivery system reforms targeting health equity. In 2014, the Institute of Medicine recommended that social and behavioral domains be incorporated into patients’ electronic health records. In 2015, these domains were codified in the *International Classification of Disease, Tenth Rev* (ICD-10) Z codes, designating “health hazards related to socioeconomic and psychosocial circumstance” inclusive of inadequate housing, unemployment, education and literacy, social environment, and financial instability. The ICD-10, which is used by all member nations of the World Health Organization, is translated into 43 languages and serves as the basis for reporting health status, mortality, and medical reimbursements.^1^ The ubiquitous use of ICD-10 codes makes the Z codes a logical mechanism for documentation and data collection on SDoH.^2^

Documentation of Z codes has increased since their introduction in October 2015.^3^ However, despite this increase, prior studies have shown that Z codes are listed in only 1–2% of inpatient charts—identifying a much smaller population than in corresponding population-level statistics for homelessness, unemployment, and low educational attainment.^3^

A high prevalence of social vulnerability among emergency department (ED) patients^4^ demands accurate documentation of SDoH. The existing literature has focused primarily on inpatient samples, single healthcare systems, or states. The frequency of Z code use specifically among ED patient populations in a national sample has not been examined. In this work, we describe the frequency of ICD-10 Z code documentation in ED charts using the Nationwide Emergency Department Sample (NEDS).^5^ We examine patient- and hospital-level characteristics associated with documentation of Z codes in EDs in the United States from 2016–2019.

**Population Health Research Capsule**

*What do we already know about this issue?*

Z codes for social determinants of health (SDoH) are documented in only 1–2% of charts—identifying a much smaller population than in corresponding population-level statistics.

*What was the research question?*

How frequently are Z codes documented in ED visits? What characteristics are associated with their use?

*What was the major finding of the study?*

While documentation of Z codes for ED visits is infrequent, it has increased from 0.65% of ED visits in 2016 to 1.17% by 2019.

*How does this improve population health?*

The high prevalence of social vulnerability among ED patients demands accurate documentation of SDoH to address drivers of health inequity.

## METHODS

This was a repeated cross-sectional analysis of ED visit data in the US from NEDS from 2016–2019.^5^ The NEDS, which is the largest all-payers claims dataset representing 900+ EDs across the US, employs complex survey weights designed to provide reliable estimates for nationwide ED visit trends. We characterized Z code use and described associations between the use of Z codes and patient- and hospital-level factors. Variables included were age, gender, race, insurance status, ED disposition, ED size, hospital urban-rural status, ownership, and US Census Region. We calculated unadjusted odds ratios for likelihood of Z code reporting for each ED visit. Additionally, we examined the most common clinical conditions, according to Clinical Classifications Software Refined (CCSR) codes, associated with patient encounters that had at least one Z code documented. The CCSR aggregates ICD-10 diagnosis codes into 530 categories for clinical conditions. Survey weights were implemented for nationally representative estimates, and standard errors were adjusted for complex sampling design. All analyses were performed in R 4.0.2 (R Foundation for Statistical Computing, Vienna, Austria).

## RESULTS

**Figure 1:**
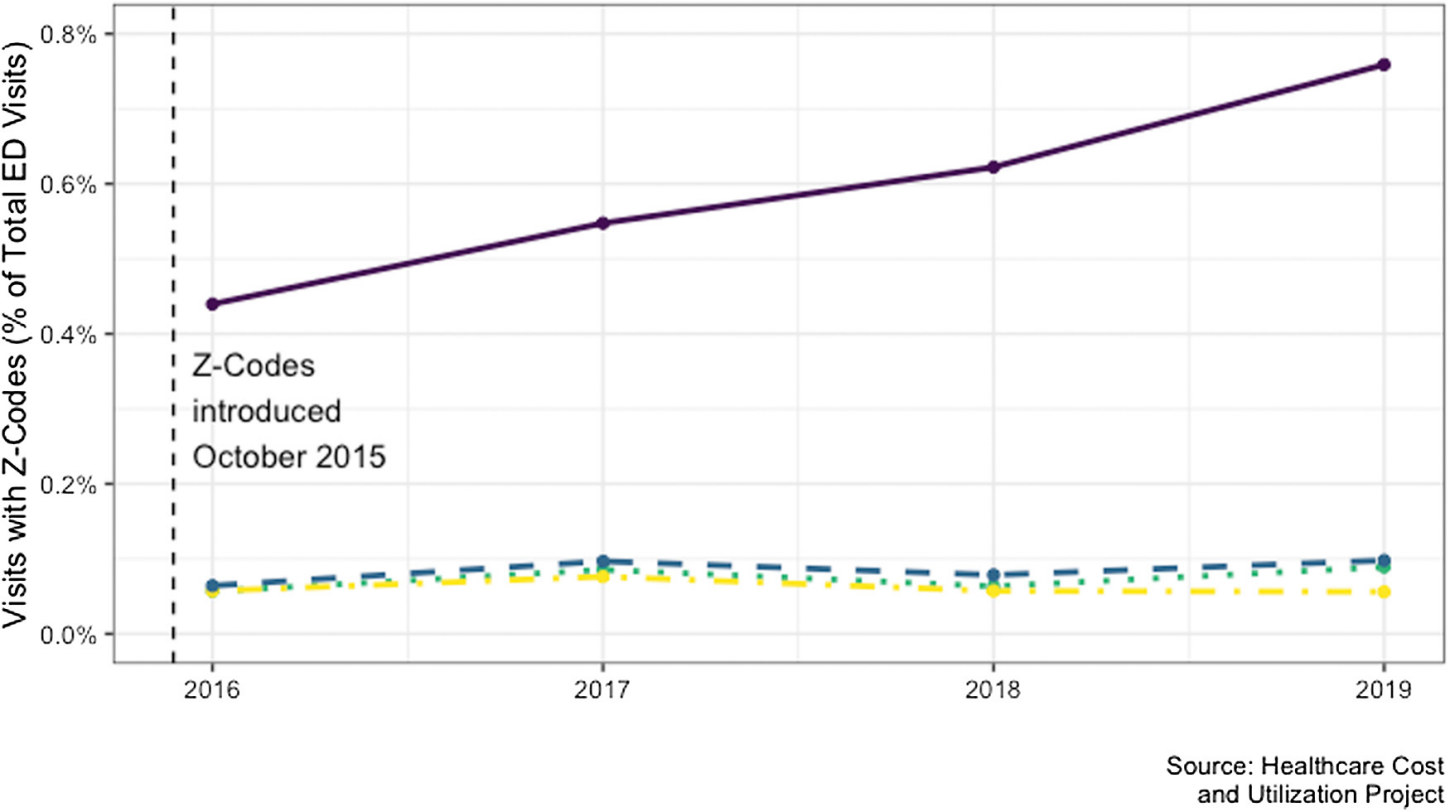
Documentation of social determinants of health among emergency department visits nationwide.

Of the approximately 140 million ED visits in each year, 0.65% had an associated Z code in 2016, rising to 1.17% by 2019. The most reported category was “problems with housing and economic circumstances,” and use of this code grew precipitously from 2016 to 2019 (from 0.44% to 0.78%) (Figure 1).

Visits were more likely to have an associated Z code for adults aged 41–64 compared to aged 19–25, male compared to female patients, those who identified their race as Black or Native American compared to those who identified White, those with Medicaid or self-pay compared to private insurance, and those who were admitted to the hospital (Table 1). Examination of hospital-level characteristics showed the Z codes were more likely to be used at larger EDs with more than 80,000 annual visits compared to smaller EDs with fewer than 20,000 visits, and academic compared to non-teaching hospitals. Z codes were less likely to be used at hospitals in micropolitan and small metropolitan areas compared to large metropolitan areas, and not-for-profit and investor-owned hospitals compared to government-run hospitals (Table 1). The most commonly associated clinical conditions were as follows: schizophrenia spectrum and other psychotic disorders (3,747; 7.4%); depressive disorder (3,521; 6.9%); and alcohol-related disorders (,479, 6.9%) (Table 2).

## DISCUSSION

Our findings demonstrate the paucity of Z code documentation^3^ specifically among ED patients, although the use of Z codes is generally uptrending. Nearly all the growth in Z code use is attributable to “issues related to housing and economic circumstances.” Z codes are more likely to be used in EDs at larger, urban, teaching hospitals and among adults age <65, male, Black, Medicaid recipient, or uninsured. Previous studies on inpatient samples have similarly found that hospitals that use Z codes are more likely to be larger, private, not-for profit, urban, teaching hospitals and that patients are more likely to be male, Medicaid recipients, or uninsured.^6^,^7^ The clinical conditions most associated with Z code use in EDs were psychiatric- and substance use-related codes. This is similar to previous work on inpatient samples that showed admissions for mental health and substance use disorders are more likely to include Z codes.^3^,^6^,^7^ Despite the uniquely high prevalence of social vulnerability among ED patients, the documentation of Z codes in the ED appears to follow a pattern similar to inpatient Z code documentation.

Prior studies have proposed that low rates of Z code use are related to clinician uncertainty on Z code relevance to a given medical encounter, ambiguity in Z codes themselves, and a lack of systematized connections to clinical screening instruments and activities.^8^ Connecting SDoH to billing structures and payment models may address some of these barriers to documentation and more substantively address the needs of patients with high social acuity.^8^ Future implementation must also be sensitive to the risk of incorporating stigmatizing language or codifying stereotypes within the medical record.^9^

## LIMITATIONS

This repeated cross-sectional analysis of NEDS has multiple limitations. First, the absence of a documented Z code for a patient encounter does not necessarily mean there was no documentation of SDoH elsewhere in the patient’s health record. However, such granular data was unavailable. Furthermore, in this analysis we were unable to characterize how strongly the medical decision-making for the clinical encounter was related to the SDoH documented in the Z codes. Finally, as there were no clinical or patient-oriented outcomes, we were unable to comment of the associations among documenting SDoH, clinical care, and outcomes.

## CONCLUSION

The ED should play a critical role in monitoring and responding to evolving health disparities by serving as a bellwether for shifts in local socioeconomic landscapes, analogous to syndromic surveillance systems where ED documentation is used to track shifting infectious disease burden.^10^ In this study we found that documentation of Z codes for ED visits is infrequent but has increased from 0.65% of ED visits in 2016 to 1.17% by 2019. Further research is warranted to better understand the drivers of clinicians’ use of Z codes and to improve on their utility. Emergency departments are uniquely positioned within the house of medicine and the social safety net to identify and address social determinants of health. Only by improved measurement can we begin to craft policy solutions to address these important drivers of health inequity.

## Figures and Tables

**Table 1:** Factors associated with use of Z codes.

	Unadjusted ORs
Visit-level characteristics	
*Primary payer (insurance status)*	
Private insurance	(ref)
Medicaid	4.23 [3.79–4.72]
Medicare	2.53 [2.28–2.81]
Self-pay	3.83 [3.38–4.34]
Other	3.86 [2.49–5.99]
*Gender*	
Female	(ref)
Male	2.21 [2.09–2.33]
*Race*	
White	(ref)
Black	1.26 [1.11–1.44]
Hispanic	0.72 [0.63–0.82]
Native American	1.53 [1.11–2.10]
Asian/Pacific Islander	0.60 [0.50–0.72]
Other	0.98 [0.78–1.23]
*Age*	
0–18	0.39 [0.34–0.45]
19–25	(ref)
26–32	1.55 [1.47–1.64]
33–40	1.91 [1.79–2.03]
41–64	2.20 [2.05–2.37]
65-	0.69 [0.62–0.76]
*Admission*	3.89 [3.48–4.35]
Hospital-level characteristics	
*Region*	
Northeast	1.17 [0.87–1.59]
Midwest	(ref)
South	1.03 [0.75–1.41]
West	2.29 [1.72–3.04]
*Urban/Rural Designation*	
Large metropolitan	(ref)
Small metropolitan	0.79 [0.64–0.98]
Micropolitan	0.33 [0.26–0.42]
*Hospital control*	
Government*	–
Private, not-for-profit	0.56 [0.41–0.78]
Private, investor-owned	0.57 [0.38–0.85]
*Teaching status*	
Metropolitan teaching	1.69 [1.39–2.06]
Metropolitan non-teaching	(ref)
*Total ED visits*	
<20,000	(ref)
20–40,000	1.44 [0.92–2.24]
40–60,000	1.72 [1.09–2.72]
60–80,000	1.75 [1.10–2.77]
80,000+	2.06 [1.30–3.24]

Binary logistic regression models were estimated using the Nationwide Emergency Department Sample 2019 data with adjustment for weighting and complex sample design and with standard errors clustered by hospital.

*ORs*, odds ratios; *ref*, reference category; *ED*, emergency department.

**Table 2:** Most commonly associated primary Clinical Classifications Software Refined codes among patients with any code for social determinants of health.

	Count	Percent	Cumulative
Schizophrenia spectrum and other psychotic disorders	3,747	7.4%	7.4%
Depressive disorders	3,521	6.9%	14.3%
Alcohol-use related disorders	3,479	6.9%	21.2%
Suicidal ideation/attempt/intentional self-harm	1,944	3.8%	25.0%
Bipolar and related disorders	1,538	3.0%	28.0%
Musculoskeletal pain, not low back pain	1,477	2.9%	30.9%
Skin and subcutaneous tissue infections	1,462	2.9%	33.8%
Septicemia	1,318	2.6%	36.4%
Nonspecific chest pain	1,212	2.4%	38.8%
Diabetes mellitus with complication	1,048	2.1%	40.9%
Trauma- and stressor-related disorders	975	1.9%	42.8%
Stimulant-use related disorders	972	1.9%	44.7%
